# Genome-Scale Reconstruction and Analysis of the Metabolic Network in the Hyperthermophilic Archaeon Sulfolobus Solfataricus

**DOI:** 10.1371/journal.pone.0043401

**Published:** 2012-08-31

**Authors:** Thomas Ulas, S. Alexander Riemer, Melanie Zaparty, Bettina Siebers, Dietmar Schomburg

**Affiliations:** 1 Department of Bioinformatics and Biochemistry, Technische Universität Braunschweig, Braunschweig, Germany; 2 Institute for Molecular and Cellular Anatomy, University of Regensburg, Regensburg, Germany; 3 Faculty of Chemistry, Biofilm Centre, Molecular Enzyme Technology and Biochemistry, University of Duisburg-Essen, Essen, Germany; Hospital for Sick Children, Canada

## Abstract

We describe the reconstruction of a genome-scale metabolic model of the crenarchaeon *Sulfolobus solfataricus*, a hyperthermoacidophilic microorganism. It grows in terrestrial volcanic hot springs with growth occurring at pH 2–4 (optimum 3.5) and a temperature of 75–80°C (optimum 80°C). The genome of *Sulfolobus solfataricus P2* contains 2,992,245 bp on a single circular chromosome and encodes 2,977 proteins and a number of RNAs. The network comprises 718 metabolic and 58 transport/exchange reactions and 705 unique metabolites, based on the annotated genome and available biochemical data. Using the model in conjunction with constraint-based methods, we simulated the metabolic fluxes induced by different environmental and genetic conditions. The predictions were compared to experimental measurements and phenotypes of *S. solfataricus*. Furthermore, the performance of the network for 35 different carbon sources known for *S. solfataricus* from the literature was simulated. Comparing the growth on different carbon sources revealed that glycerol is the carbon source with the highest biomass flux per imported carbon atom (75% higher than glucose). Experimental data was also used to fit the model to phenotypic observations. In addition to the commonly known heterotrophic growth of *S. solfataricus*, the crenarchaeon is also able to grow autotrophically using the hydroxypropionate-hydroxybutyrate cycle for bicarbonate fixation. We integrated this pathway into our model and compared bicarbonate fixation with growth on glucose as sole carbon source. Finally, we tested the robustness of the metabolism with respect to gene deletions using the method of Minimization of Metabolic Adjustment (MOMA), which predicted that 18% of all possible single gene deletions would be lethal for the organism.

## Introduction

In recent years an increase of the use of genome-scale computer models for the reconstruction and prediction of cellular metabolic properties can be observed. Metabolic reconstruction starts with a full genome annotation of a particular organism, in particular the prediction of enzymes and transporters, and the addition of enzyme-catalyzed reactions. This allows an in-depth insight into the metabolic capacities of the organism. With growing availability of genomes across the three domains of life, genome-scale reconstructions of metabolic networks are now available for a number of organisms such as *Escherichia coli* (Bacteria) [1], *Methanosarcina acetivorans* (Archaea) [2], and *Arabidopsis thaliana* (Eukaryota) [3]. Metabolic reconstructions can be analyzed using constraint-based methods, where physiological constraints, such as uptake rates, and chemical constraints, such as reversibility of reactions, represent the constraints of the model [4]. One method for analyzing a constraint-based model is flux balance analysis (FBA) [5]. FBA determines the steady-state flux distribution of a constraint-based network by maximizing an objective function, most commonly the growth rate for a given carbon source intake. A special feature of FBA is the possibility to get information about the flux distribution or the variability of the network under certain conditions. Further examinations of the model like *in-silico* knockout studies and prediction of growth on different carbon sources can give important new insights, which can be used for metabolic engineering. One example is the optimization of L-lysine production in *Corynebacterium glutamicum* [6]. To date there are only two models of archaeal organisms available (*Methanosarcina barkeri* [7] and *Methanosarcina acetivorans* [2]), in contrast to the numerous published models of Bacteria and Eukaryota (http://gcrg.ucsd.edu). Both *M. barkeri* and *M. acetivorans* are mesophilic anaerobic methanogens, which are very closely related to each other (same genus) and belong to the phylum Euryarchaeota. Our model of S. solfataricus covers the other major branch of Archaea, the Crenarchaeota, and therefore represents a considerable gain of knowledge about the Archaea. The Archaea are of special interest because most culturable species are able to survive in extreme environments. The versatility of archaeal (hyper)thermophiles ranges from survival in the deep sea, in marine or terrestrial environments with temperatures over 100°C (>60°C up to 116°C) to very acidic or alkaline milieu. As an interesting and experimentally characterized representative of the Archaea, *Sulfolobus solfataricus* was modeled in this work.

*S. solfataricus* is a hyperthermoacidophilic archaeon and represents one of the best-studied hyperthermoacidophilic organisms within the phylum Crenarchaeota. It colonizes terrestrial volcanic hot springs and was originally isolated from the Pisciarelli solfataric field near Naples, Italy. *S. solfataricus* has an optimal growth at 80°C (60–92°C) and pH 2–4 [8], [9]. The genus *Sulfolobus* is able to oxidize sulfur, forming sulfate. *S. solfataricus* is strictly aerobic and grows either heterotrophically or uses cellular respiration with oxygen acting as the final electron acceptor for autotrophic growth. It has a number of transport systems for the uptake of different organic compounds and energy sources such as sugars (e.g. glucose and galactose) and amino acids (e.g. alanine, aspartate, and glutamate) [10]. *S. solfataricus* is also able to grow chemolithoautotrophically using bicarbonate fixation [11]–[13]. The first sequenced genome of *Sulfolobus*, *S. solfataricus* P2, was published in 2001 [14]. It contains 2,992,245 bp on a single circular chromosome [15] encoding 2,977 proteins. About one third of the genes have no known homologs in other sequenced genomes. *Sulfolobus* is of high interest for industry and biotechnology because of its broad physiological versatility – e.g. it exhibits the ability to grow on phenol [16] – and because of the unique properties of its thermostable proteins (“extremozymes”). In addition, *S. solfataricus* is a model organism for research on the mechanisms of DNA replication, DNA processing, chromosomal integration, transcription, translation, and the cell cycle [17].

## Results and Discussion

### Reconstruction of the S. solfataricus model

The initial model was created pathway by pathway from the genome annotation and manually completed to allow the production of all biomass constituents. This metabolic reconstruction of *S. solfataricus* was based on published annotations of the *S. solfataricus* P2 genome, different biochemical databases including KEGG [18], MetaCyc [19], BRENDA [20], *Sulfolobus*-specific literature, and experimental data generated in our lab. Especially the biosynthetic pathways of the unique coenzymes sulfopterin, caldariellaquinone and sulfolobusquinone could only be obtained from *Sulfolobus*-specific literature. The model was generated and improved using an iterative model reconstruction process (see Material and Methods and Figure S1). At the end of the manual reconstruction phase, the model included 581 reactions (including 112 gap-filling reactions), 468 EC numbers and 397 genes. This first model was fully functional with respect to the production of all biomass components.

### Automatic addition of remaining annotated enzymes

After the manual model reconstruction process, an automated completion step was performed. In this procedure, any remaining EC number was added to the final model. Only EC numbers with a high reliability were integrated (either predicted by all of the databases KEGG, MetaCyc, and BRENDA or having an E-value below 10^−40^, see Material and Methods – Genome annotation).

Out of the 2,977 [14] identified genes, 944 genes belonging to 586 different enzyme classes were predicted with a high reliability. After removal of the genes and EC numbers from the first manually-created model, 366 genes with a high reliability corresponding to 228 unique enzyme classes remained. Out of those 228 remaining EC numbers, 33 had an incomplete enzyme classification. 115 of the remaining 195 EC numbers were either associated with generic intermediates like “protein” or “an alcohol” or with polymerization like “DNA”, “RNA” etc. Finally, the 137 reactions catalyzed by the remaining 80 enzyme classes (118 unique genes) were additionally integrated into the model. 58 of these 137 reactions are isolated reactions and have no connection to the remaining network.

### Characterization of the metabolic model

The resulting metabolic network consists of 706 unique metabolites connected by 718 metabolic reactions, one biomass reaction, and 58 transport/exchange reactions. 112 reactions were manually added to fill gaps in the reconstructed network after the initial usage of enzyme functions from the genome annotation. The model iTU515 of *S. solfataricus* covers 515 genes involved in 606 gene-related metabolic reactions. Altogether 134 reactions were set irreversible, because they were marked as irreversible in BRENDA; 77 of them are hydrolase reactions, 57 others. The basic characteristics and comparisons with other metabolic reconstructions are presented in Table 1.

**Table 1:** Comparison of the properties of the metabolic reconstructions of *S. solfataricus*, *M. barkeri*, *C. salexigens*, *M. tuberculosis*, and *E. coli*.

Reconstructed Organism	S. solfataricus iTU515	M. barkeri iAF692	C. salexigens iOA584	M. tuberculosis iNJ661	E. coli iAF1260
Genome size of organism	2.9 MB	4.8 MB	3.7 MB	4.4 MB	4.6 MB
Total genes in organism (ORFs)	2977	5072	3352	3989	4464
SKI value	0,39	0,076	0,008	11,21	62,05
Genes (% of genome) in genome-scale model	515 (17%)	542 (11%)	558 (17%)	661 (17%)	1260 (28%)
Reactions in genome-scale model	718	619	876	939	2077
Biochemical evidence	299	-	-	-	-
High evidence (functional annotation from probable homologs)	103	-	-	-	-
Medium evidence (functional annotation from average homologs)	204		-	-	-
Gap filling (low evidence with score <6 and non-gene-related reactions)	112 (16%)	110 (18%)	20 (2%)	116 (16%)	158 (8%)
Reactions catalyzed by defined gene products	606	509	856	723	1919
Active reactions during growth on glucose	352	-	-	-	412
Transport and exchange reactions	58	88	510	88	304
Metabolites in genome-scale model	705	558	920	828	1039

### Properties and comparison of iTU515 with published metabolic networks

The properties of our metabolic network were compared with the properties of published reconstructions of *Methanosarcina barkeri* (iAF692, [7]), *Chromohalobacter salexigens* (iOA584, [21]), *Mycobacterium tuberculosis* (iNJ661, [22]) and *Escherichia coli* (iAF1260, [1]) (Table 1). So far two closely related archaeal models have been published (*M. barkeri* and *M. acetivorans*). We included a detailed comparison of our model with *M. barkeri*, which is the most comparable model organism to *S. solfataricus*. The percentage of included genes of iTU515 is smaller than in the *E. coli* model but higher compared to the other published archaeal model of *M. barkeri* and the model of the extremophile *C. salexigens*. Furthermore, the species knowledge index (SKI) [23], which relates the number of PubMed abstracts of an organism to its number of ORFs, is much lower for *S. solfataricus* (0,39) compared to the other reconstructions except for *M. barkeri* (0,076) and *C. salexigens* (0.008). Compared with the bacterial *M. tuberculosis* model, our ratio of included genes is very similar, although *M. tuberculosis* has a much higher SKI value. The number of included transport reactions is lower than in *M. barkeri*, *M. tuberculosis*, *C. salexigens*, and *E. coli*, indicating a lower versatility of *S. solfataricus*, smaller genome size, or the lack of information for *S. solfataricus* due to its low SKI. While the total number of reactions differs strongly between iTU515 and the *E. coli* model iAF1260, the number of active reactions is similar (iTU515: 352, iAF1260: 412 (based on published flux distribution)). Active reactions are those with a non-zero flux, including metabolic, transport/exchange and biomass reactions. In summary, this comparison indicates that the properties of iTU515 are comparable with other high-quality network reconstructions, especially considering the relative scarcity of available information in the literature.

The reactions included in iTU515 were divided into 65 specific pathways and subsystems based on their functional role. The pathways of purine and pyrimidine metabolism were found to contain the highest numbers of reactions (48 and 54 reactions, respectively). More than one-third of the other pathways are related to the catabolism of various compounds, which reflects the physiological ability of *S. solfataricus* to use a variety of compounds as carbon and energy sources.

### General properties of the model-based simulations of *S. solfataricus*

After the reconstruction of S. solfataricus metabolism, the model was checked for the ability to accurately predict the growth yield, which is one of the basic properties of the modeled organism. In order to simulate growth on a single carbon source, four parameters have to be experimentally determined or defined: The nutrient uptake rate, the CO2 production rate (respiration), the P/O ratio, and the maintenance energy demand.

The nutrient uptake rate and the CO_2_ production rate are necessary for the analysis of the network to predict the efficiency of biomass production, i.e. the ratio between integrated and respirated carbon atoms. In this context it is important to know if any other metabolites are secreted into the medium. The respiration efficiency of the model is described as the P/O ratio. The P/O ratio represents the number of ATP molecules produced from the reduction of one oxygen atom during respiration. The P/O ratio was set to 0.5 because *Sulfolobus* uses the unusual archaea-specific cytochrome complexes SoxABCD and SoxEFGHIM [24], which have a low oxidation efficiency [25]. The maintenance energy is composed of the growth-associated (GAM) and the non-growth-associated maintenance (NGAM) energy [26], [27], which represent the energy “costs of living” of the cell. The GAM cost accounts for the energy (ATP) necessary for mechanisms like replication, transcription, and translation and is integrated into the biomass objective function. The NGAM represents the requirements of the cell to maintain, e.g., its membrane potential and turgor pressure and the energy necessary for processes such as protein refolding or DNA repair [1]. It is represented as an ATP hydrolysis reaction (ATP+H_2_O→ADP+Pi). The GAM value was determined from the genomic summary of S. solfataricus P2 from the Comprehensive Microbial Resource website (http://cmr.jcvi.org) [28] and computed afterward [29]. The calculated value of 24.86 mmol ATP g_DW_^−1^ is between the values published for different E. coli models and similar to the GAM value of 29.2 mmol ATP g_DW_^−1^, which was published for *Corynebacterium glutamicum* [30].

**Figure 1:**
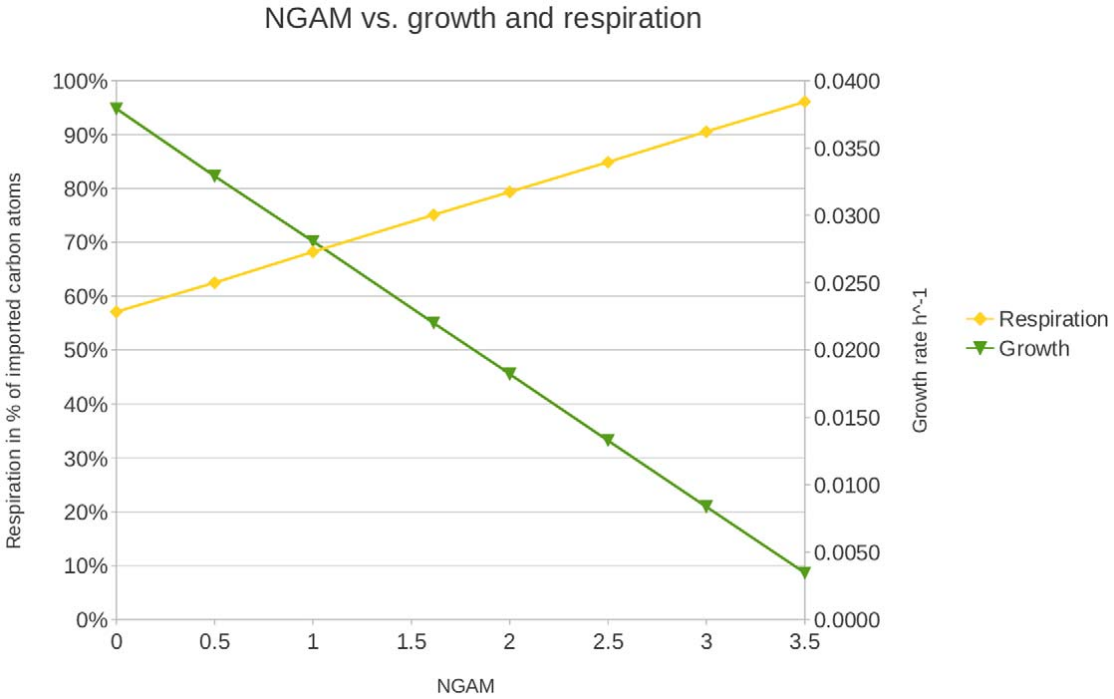
Diagram of the influence of non-growth-associated maintenance (NGAM) energy on the biomass flux and the respiration in *S. solfataricus*.

Subsequently, the effects of changes in the GAM on the in-silico growth yield were tested. A twofold increase led to a decrease of the biomass flux by only 5–15%. The NGAM was set to 1.9 mmol ATP g_DW_^−1^ h^−1^, because this value resulted in a carbon usage ratio of 25%, which was determined in our laboratory [31]. The NGAM value had a stronger effect on the whole system. An increase from 1 mmol ATP g_DW_^−1^ h^−1^ to 2 mmol ATP g_DW_^−1^ h^−1^ decreased the biomass flux by 35% (Figure 1).

### Growth on glucose

*S. solfataricus*, like all Sulfolobales, has an incomplete Embden-Meyerhof-Parnas pathway, with phosphofructokinase missing [32]. However, *S. solfataricus* utilizes a branched Entner-Doudoroff pathway. Glucose was used as main carbon source to investigate the properties of iTU515 and to evaluate and verify the reconstructed network. The glucose uptake rate was determined in the laboratory to be 0.55 mmol g_DW_^−1^ h^−1^ at 80°C [31] and was included in the model. We compared the *in-silico* predicted growth with the experimental data to get a direct evaluation of the potential of iTU515. In agreement with experimental results [31], the calculated growth rate of 0.0222 h^−1^ is very small compared to Bacteria. In comparison, the growth rate of *E. coli* is 0.68 h^−1^ at a glucose uptake rate of 10.5 mmol g_DW_^−1^ h^−1^ [33]. The relative biomass yield of glucose is 25% and thus much lower compared to *E. coli* with 42% [34]. One possible explanation could be the low P/O ratio of 0.5 (*E. coli*: 1.5 [33]), caused by the low electron transport chain efficiency and the membrane, which was shown to be leaky for protons [35]. Since no exact value is known for *Sulfolobus*, a general value for the P/O ratio of Archaea had to be taken from the literature [25]. Other possible explanations for the low biomass yield in comparison to *E. coli* could be the inefficiency of metabolism at high temperatures due to the instability of metabolites [36] and the possibility that *S. solfataricus* does not use all of the imported glucose for biomass formation and respiration. Recent literature studies described a carbon recovery between 72% and 83% [37], [38], where only 25% of the imported carbon atoms are used for biomass formation. One possible explanation for this discrepancy is that *S. solfataricus* is known to produce exopolysaccharides (EPS) [39], [40]. Glucose, mannose, galactose, and N-sulfo-D-glucosamine are assumed to be the minimal components of the described EPS. Based on this, we assume an integration of 25% of the imported carbon atoms into biomass, where less than 1% consist of intracellular glycogen [41] and trehalose, and a usage of 75% for respiration [42].

**Figure 2:**
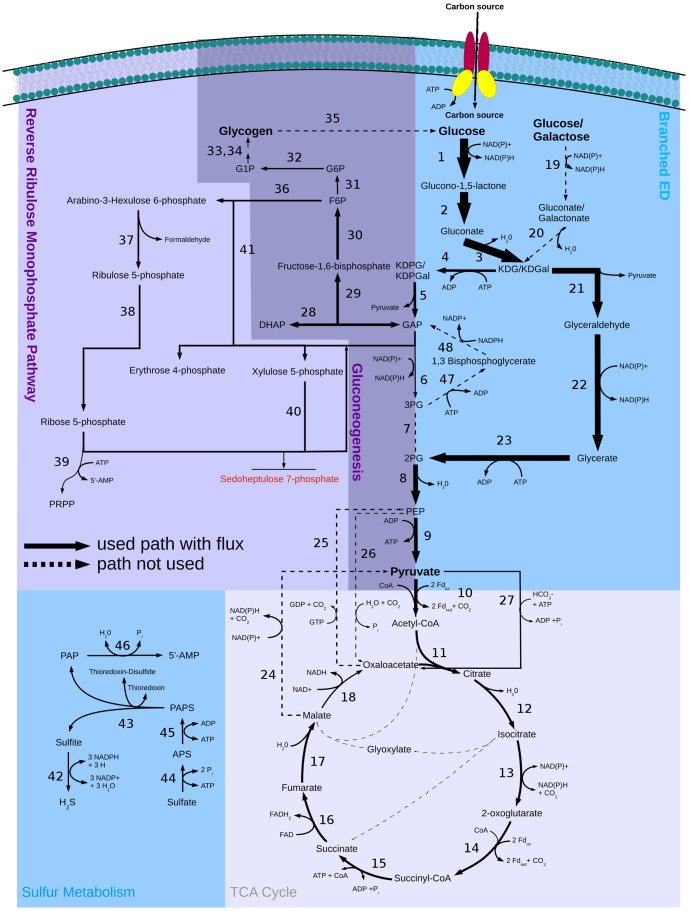
Central carbon metabolism in *S. solfataricus* with flux distribution for growth on glucose. Solid arrows show the used pathways and their directions. Dashed arrows show available but not used pathways. Arrow size represents predicted flux though the pathways. Enzymes in this pathway: (1) glucose 1-dehydrogenase, (2) gluconolactonase, (3) gluconate dehydratase (4) 2-dehydro-3-deoxygluconokinase, (5) 2-dehydro-3-deoxy-phosphogluconate aldolase, (6) glyceraldehyde-3-phosphate dehydrogenase, (7) phosphoglycerate mutase, (8) phosphopyruvate hydratase, (9) pyruvate kinase, (10) pyruvate synthase, (11) citrate (Si)-synthase, (12) aconitate hydratase, (13) isocitrate dehydrogenase, (14) 2-oxoglutarate synthase, (15) succinate-CoA ligase, (16) succinate dehydrogenase, (17) fumarate hydratase, (18) malate dehydrogenase, (19) glucose 1-dehydrogenase, (20) galactonate dehydratase, (21) 2-keto-3-deoxygluconate aldolase, (22) aldehyde dehydrogenase, (23) glycerate kinase, (24) malate dehydrogenase, (25) phosphoenolpyruvate carboxykinase (GTP), (26) phosphoenolpyruvate carboxylase, (27) pyruvate carboxylase, (28) triose-phosphate isomerase, (29) fructose-bisphosphate aldolase, (30) fructose-bisphosphatase, (31) glucose-6-phosphate isomerase, (32) beta-phosphoglucomutase, (33) glucose-1-phosphate adenylyltransferase, (34) starch synthase, (35) glucan 1,4-alpha-glucosidase, (36) 6-phospho-3-hexuloisomerase, (37) 3-hexulose-6-phosphate synthase, (38) ribose-5-phosphate isomerase, (39) ribose-phosphate diphosphokinase, (40) transketolase, (41) transketolase, (42) sulfite reductase, (43) phosphoadenylyl-sulfate reductase, (44) sulfate adenylyltransferase, (45) adenylyl-sulfate kinase, (46) 3′(2′),5′-bisphosphate nucleotidase, (47) phosphoglycerate kinase, (48) glyceraldehyde-3-phosphate dehydrogenase.

Furthermore, *S. solfataricus* has a reverse ribulose-monophosphate pathway instead of the pentose phosphate pathway (Figure 2; upper left part). Due to the fact that *S. solfataricus* is missing transaldolase, it was not possible to find a reaction to metabolize the accumulating sedoheptulose 7-phosphate produced by transketolase (Figure 2: (40)). In order to reach a working model, either a putative transaldolase enzyme or a transport reaction to export the excess sedoheptulose 7-phosphate had to be added to the model. As the leak flux corresponds to a loss of carbon that is less than 3% of the imported carbon atoms (glucose) and this alternative did not influence the FBA results in any significant way, we decided to include the transporter. This point has to be clarified in the future. *S. solfataricus* imports glucose via an ABC transporter and metabolizes it via the modified Entner-Doudoroff pathway, where glucose can be metabolized via the non-phosphorylative or the semi-phosphorylative branch (Figure 2). In the simulation, 22% of the glucose is phosphorylated via the semi-phosphorylative branch leading to the reverse ribulose-monophosphate pathway and to glycogen and trehalose formation. The remaining 78% of the glucose is metabolized to pyruvate via the non-phosphorylative branch, followed by the tricarboxylic acid cycle (TCA cycle). The TCA cycle is then refilled by one of the anaplerotic reactions (phosphoenolpyruvate+CO_2_+H_2_O→oxaloacetate+Pi).

### Optimal flux variability analysis (FVA)

The optimal growth rate can often be achieved through a number of alternative flux distributions. Therefore, flux variability analysis (FVA) was used to explore the solution space. This method computes the allowed values for each flux keeping the optimal value of the biomass yield.

The analysis showed that 57 of the 352 active reactions during growth on glucose show a variability of more than 100% of their predicted value, and another 1 showed a variability between 30 and 100%. 197 reactions with a predicted flux of zero could assume non-zero values in an alternative optimal solution. The fluxes of 294 reactions are fixed by the optimal conditions, representing 83% of the network. Such a high value indicates a well-defined network. None of the active reactions had a higher carbon flux than the glucose uptake rate.

The analysis of flux variabilities showed candidates for reactions involved in stoichiometrically balanced cycles [29]. For the results of FBA, these cycles do not create problems, because in our implementation of FBA, non-productive fluxes, i.e. fluxes that do not contribute to biomass production, do not occur. These reactions were found to have a large variability because they can be replaced by a combination of other reaction. Stoichiometrically balanced cycles were identified by our in-house software xeledon (Rex and Schomburg, to be published) but not removed because they were frequently found to be reversible.

The highest flux variabilities were observed for the semi-phosphorylative and the non-phosphorylative branches of the Entner-Doudoroff pathway. The yield of energy carriers from the semi-phosphorylative and from the non-phosphorylative branch is one ATP for each branch metabolizing one molecule of glucose to two molecules of pyruvate. Because both branches are energetically equivalent, they can be used alternatively by *S. solfataricus*. The FVA calculations show no flexibility for the reverse ribulose-monophosphate pathway, which reflects the importance of this part of the network. In contrast, the TCA cycle shows a greater variability. FVA calculations suggest that the glyoxylate shunt could be used instead of the normal TCA cycle without any loss of growth yield. According to the literature, the glyoxylate shunt is inactivated via catabolic repression in many bacteria grown on glucose [43], and also in *S. solfataricus* [44], [45]. FVA revealed that anaplerotic refilling of the TCA cycle can proceed either using pyruvate carboxylase integrating hydrogen carbonate (Figure 2: (27)) or using phosphoenolpyruvate carboxylase integrating carbon dioxide (Figure 2: (26)). An increased variability was also calculated for the final biosynthesis steps of compounds like histidine, tryptophan, alanine, and glutamate because these can be produced via more than one pathway.

79 of 834 reactions showed a flux variability of more than 0.0001 mmol g_DW_^−1^ h^−1^ (glucose uptake rate 0.55 mmol g_DW_^−1^ h^−1^) with 471 reactions having a flexibility between 10^−6^ and 10^−22^ mmol g_DW_^−1^ h^−1^ and 284 reactions having zero variability.

### Suboptimal FVA

In a second analysis, the influence of suboptimal adaption to the environment on flux variability was assessed. To this goal an extended flux variability analysis was performed with the requirement that at least 95% of the optimal biomass production be achieved.

The results showed an increase of the variability of more than 100% for 126 (optimal FVA: 57) of the 352 active reactions. Another 17 reactions (optimal FVA: 1) had a variability between 30 and 100%. 197 reactions with a predicted flux of zero (optimal FVA: 197) could assume non-zero values in an alternative suboptimal solution, and 209 reactions (optimal FVA: 294) have fixed flux values in this case.

The results show a very similar flexibility of the Entner-Doudoroff pathway as described for optimal FVA before. In contrast to this, the reverse ribulose-monophosphate pathway shows a slightly higher variability than before. The overall flexibility of the network increased from 79 to 352 reactions with a flux variability of more than 0.0001 mmol g_DW_^−1^ h^−1^. The flux variability of the transport and degradation reactions remained unaffected. In summary, increasing the tolerance of the optimal FBA solutions by 5% increased the flux variability of the whole network on average by 345%. The comparison of the optimal and suboptimal results indicates that there exist multiple suboptimal solutions across the network.

### Modified Growth on glucose producing exopolysaccharides (EPS)

As described before, there is evidence that *S. solfataricus* uses between 17% and 28% of the imported carbon for the production of exopolysaccharides under certain conditions [39], [40]. We assumed a mean value of 22.5% for the production of exopolysaccharides. Within the model the EPS-forming reaction is represented as the sum of the molar ratios of different sugars producing one “unit” of EPS, which is exported (1.2 glucose+mannose+0.18 N-sulfo-D-glucosamine+0.13 galactose→EPS+H_2_O [39], [40]). The EPS flux was set to 0.049 mmol g_DW_^−1^ h^−1^ corresponding to 22.5% EPS of the imported carbon atoms from 0.55 mmol g_DW_^−1^ h^−1^ glucose. The NGAM value and the glucose uptake rate were kept at the same values as during glucose growth without EPS production (NGAM: 1.9 mmol ATP g_DW_^−1^ h^−1^; glucose uptake rate: 0.55 mmol g_DW_^−1^ h^−1^).

Only those flux differences were counted to be significant where the two variation intervals as determined by optimal FVA do not overlap (Material and Methods – FVA). The only significant differences can be found in the pathways leading to the production of the EPS compounds glucose, mannose, N-sulfo-D-glucosamine, and galactose (results not shown). The predicted biomass flux was decreased during growth on glucose producing EPS (0.0115 h^−1^ as compared to 0.0222 h^−1^ without EPS). The loss of energy, caused by the formation of energy-rich EPS also leads to a decrease of the ratio between biomass and CO_2_ formation (1∶3 in the non-EPS scenario vs. approximately 1∶4 in the EPS scenario).

### Autotrophic carbon fixation in *Sulfolobus solfataricus*

**Figure 3:**
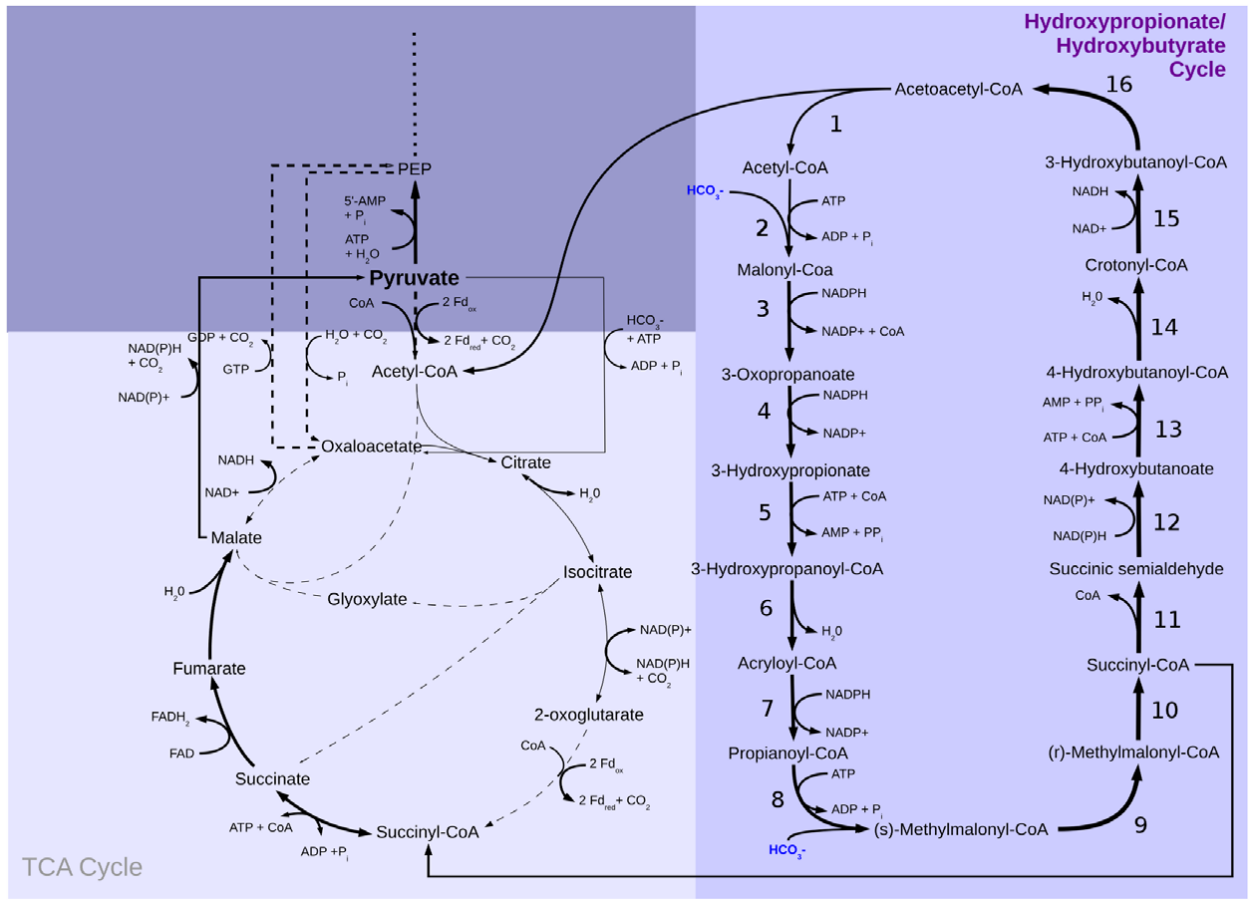
Hydroxypropionate-hydroxybutyrate cycle and tricarboxylic acid cycle in *S. solfataricus* with flux distribution for growth on HCO_3_^−^. Solid arrows show the used pathways and their directions. Dashed arrows show available but not used pathways. Arrow size represents predicted flux though the pathways. Enzymes in the hydroxypropionate-hydroxybutyrate cycle are: (1) acetyl-CoA C-acetyltransferase, (2) acetyl-CoA carboxylase, (3) malonyl-CoA reductase (NADPH), (4) malonic semialdehyde reductase (NADPH), (5) 3-hydroxypropionate-CoA ligase, (6) 3-hydroxypropionyl-CoA dehydratase, (7) acryloyl-CoA reductase (NADPH), (8) propionyl-CoA carboxylase, (9) methylmalonyl-CoA epimerase, (10) methylmalonyl-CoA mutase, (11) succinyl-CoA reductase, (12) succinic semialdehyde reductase (NADPH), (13) 4-hydroxybutyrate-CoA ligase, (14) 4-hydroxybutyryl-CoA dehydratase, (15) enoyl-CoA hydratase, (16) (S)-3-hydroxybutyryl-CoA dehydrogenase (NAD^+^).

*Sulfolobus sp.* VE 6, which is closely related to *S. solfataricus P2*, is able to grow chemolithoautotrophically using bicarbonate fixation via the hydroxypropionate-hydroxybutyrate cycle [11], [12], [46] under aerobic conditions [13] (Figure 3). 11 of 16 necessary enzymes for the hydroxypropionate-hydroxybutyrate cycle were also predicted for *S. solfataricus P2*. For the missing five enzymes, a BLAST search with known enzyme sequences on the *S. solfataricus* genome revealed very high homology with E-values of 0.0 for three of them (sequence identities between 74% and 81%) and E-values of 10^−11^ and 10^−24^ for the other two (sequence identities of 30% and 47%, respectively), which gives additional theoretical support. In particular, the key enzymes acetyl-CoA carboxylase (E-value: 10^−127^; Figure 3: (2)), propionyl-CoA carboxylase (E-value<10^−179^; Figure 3: (8)), methylmalonyl-CoA mutase (E-value<10^−179^; Figure 3: (10)) and enoyl-CoA hydratase (E-value: 10^−150^; Figure 3: (15)) were identified in the genome. Therefore, we included the whole cycle into our P2 model and modeled the flux distribution, but experimental validation is required. In the process of carbon fixation, two molecules of hydrogen carbonate are incorporated into acetyl-CoA giving succinyl-CoA. Succinyl-CoA is partially imported into the TCA cycle, and the rest is converted into two acetyl-CoA molecules, where one is used for the next turn of the hydroxypropionate-hydroxybutyrate cycle [47], and the other is redirected into the TCA cycle for the reduction of NAD^+^, NADP^+^, or FAD^+^ and for the production of biomass precursors. Import of hydrogen sulfide as reducing agent [48] proved to be necessary for chemolithoautotrophic growth, and the sulfide is used as an alternative electron donor in energy metabolism. For comparability, the NGAM value was kept at the same value as during growth on glucose (1.9 mmol ATP g_DW_^−1^ h^−1^) in our simulation of autotrophic carbon fixation (CO_2_-fixation scenario). To ensure the same carbon uptake as during growth on glucose, the hydrogen carbonate uptake rate was set to 3.3 mmol g_DW_^−1^ h^−1^.

**Figure 4:**
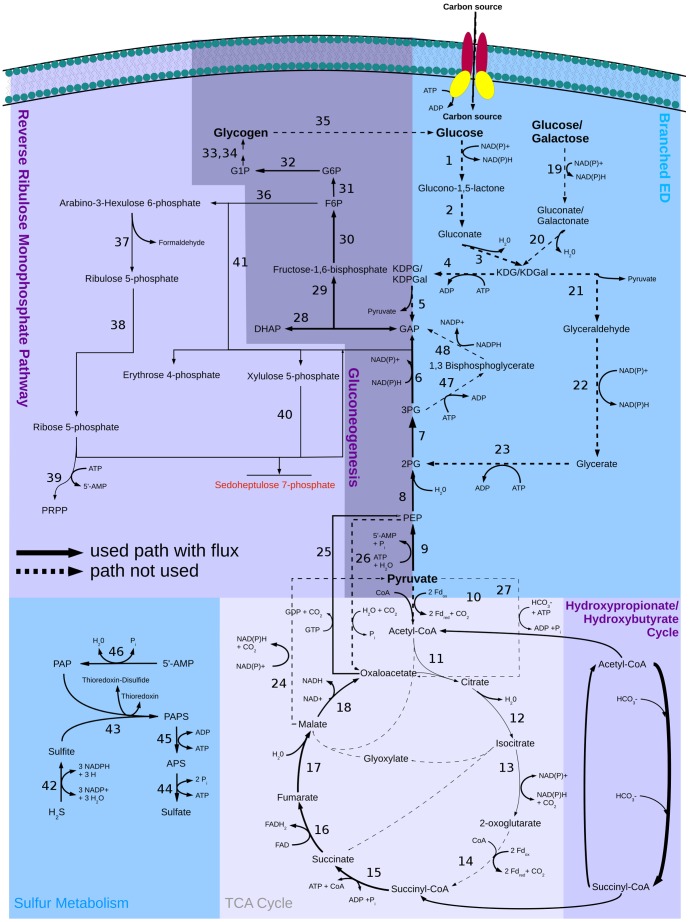
Central carbon metabolism in *S. solfataricus* with flux distribution for growth on HCO_3_^−^. Solid arrows show the used pathways and their directions. Dashed arrows show available but not used pathways. Arrow size represents predicted flux though the pathways. Enzymes in this pathway: (1) glucose 1-dehydrogenase, (2) gluconolactonase, (3) gluconate dehydratase (4) 2-dehydro-3-deoxygluconokinase, (5) 2-dehydro-3-deoxy-phosphogluconate aldolase, (6) glyceraldehyde-3-phosphate dehydrogenase, (7) phosphoglycerate mutase, (8) phosphopyruvate hydratase, (9) pyruvate kinase, (10) pyruvate synthase, (11) citrate (Si)-synthase, (12) aconitate hydratase, (13) isocitrate dehydrogenase, (14) 2-oxoglutarate synthase, (15) succinate-CoA ligase, (16) succinate dehydrogenase, (17) fumarate hydratase, (18) malate dehydrogenase, (19) glucose 1-dehydrogenase, (20) galactonate dehydratase, (21) 2-keto-3-deoxygluconate aldolase, (22) aldehyde dehydrogenase, (23) glycerate kinase, (24) malate dehydrogenase, (25) phosphoenolpyruvate carboxykinase (GTP), (26) phosphoenolpyruvate carboxylase, ((27) pyruvate carboxylase, (28) triose-phosphate isomerase, (29) fructose-bisphosphate aldolase, (30) fructose-bisphosphatase, (31) glucose-6-phosphate isomerase, (32) beta-phosphoglucomutase, (33) glucose-1-phosphate adenylyltransferase, (34) starch synthase, (35) glucan 1,4-alpha-glucosidase, (36) 6-phospho-3-hexuloisomerase, (37) 3-hexulose-6-phosphate synthase, (38) ribose-5-phosphate isomerase, (39) ribose-phosphate diphosphokinase, (40) transketolase, (41) transketolase, (42) sulfite reductase, (43) phosphoadenylyl-sulfate reductase, (44) sulfate adenylyltransferase, (45) adenylyl-sulfate kinase, (46) 3′(2′),5′-bisphosphate nucleotidase, (47) phosphoglycerate kinase, (48) glyceraldehyde-3-phosphate dehydrogenase.

**Figure 5:**
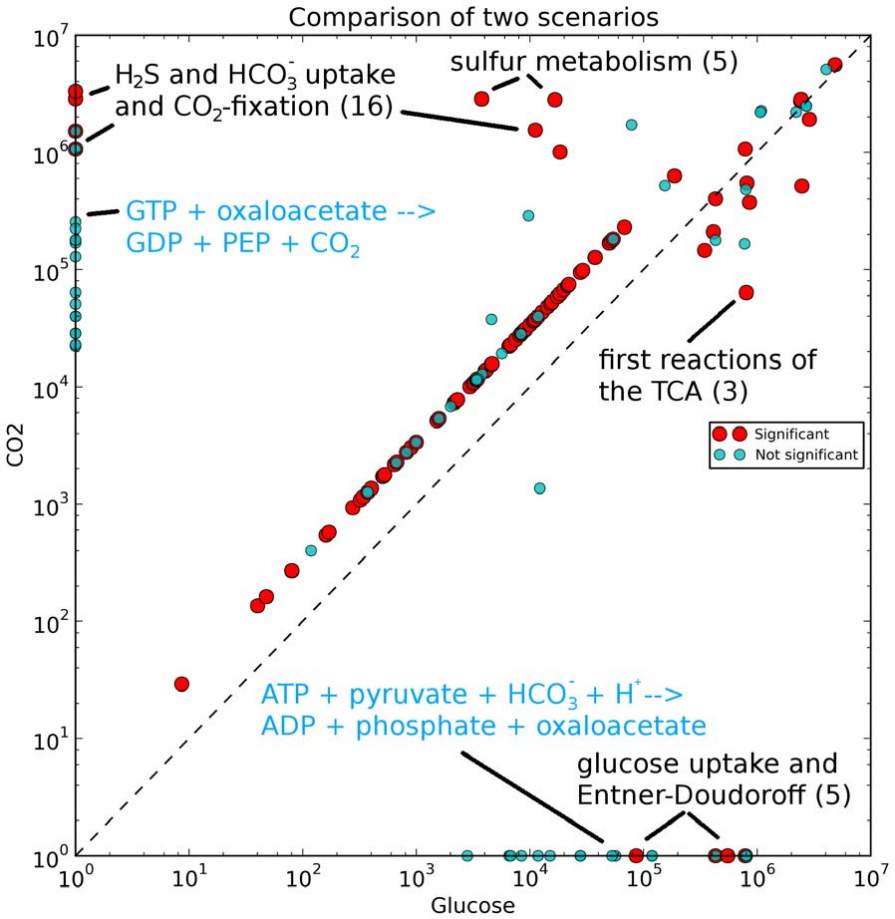
Scatter plot of the flux distributions during growth on glucose and the carbon fixation scenario. Red dots show significant differences, and blue dots show not significant differences between both scenarios. Blue labels describe not significant differences. The numbers indicate the number of involved reactions.

As expected, the Entner-Doudoroff pathway was inactive, and the hydroxypropionate-hydroxybutyrate cycle was active (Figure 4), with flux through gluconeogenesis ensuring the production of necessary intermediates for the reverse ribulose-monophosphate pathway (CO_2_-fixation scenario). The only significant differences are the sulfur metabolism with the oxidation of hydrogen sulfide (Figure 4 (42–46)), the active hydroxypropionate-hydroxybutyrate cycle (Figure 3 (1–16)), and the inactive Entner-Doudoroff pathway (Figure 4 (1–7,21–23)) (red circles; Figure 5) in the CO_2_-fixation scenario. Other significant differences are the decreased fluxes through the first three reactions of the TCA cycle (Figure 4 (11–13)) converting acetyl-CoA to 2-oxoglutarate in the bicarbonate fixation, because hydrogen carbonate is reduced partially to succinyl-CoA, which enters the TCA cycle. The small flux to 2-oxoglutarate during bicarbonate fixation ensures the production of, e.g., amino acids. As shown in the figure, a reaction forming phosphoenolpyruvate (GTP+oxaloacetate→GDP+phosphoenolpyruvate+CO_2_) is essential for the CO_2_ scenario, whereas it is dispensable for growth on glucose. On the other hand, the anaplerotic reaction (ATP+pyruvate+HCO_3_^−^+H^+^→ADP+phosphate+oxaloacetate) shown in the figures is indispensable for growth on glucose.

Since the uptake rate for hydrogen sulfide is not known during bicarbonate fixation, no constraint was placed on this reaction. This had the effect that most of the energy necessary for growth is taken from the oxidation of hydrogen sulfide in the optimal solution, because energy production by hydrogen sulfide oxidation is not associated with loss of carbon atoms. The calculated biomass flux is 0.0745 h^−1^ and thus much higher than the biomass flux calculated for growth on glucose (0.0222 h^−1^) (shifted diagonal line in the direction of bicarbonate fixation) (red circles; Figure 5). However, this difference in biomass flux is strongly dependent on the hydrogen sulfide uptake rate, which becomes evident by considering the cost of producing one acetyl-CoA from hydrogen carbonate and CoA (4 ATP and 3 NAD(P)H). The hydrogen sulfide flux calculated by FBA was 3.1 mmol g_DW_^−1^ h^−1^. Constraining this flux to 2.15 mmol g_DW_^−1^ h^−1^ halved the biomass yield (not shown), highlighting the importance of this parameter.

### Growth on phenol

**Figure 6:**
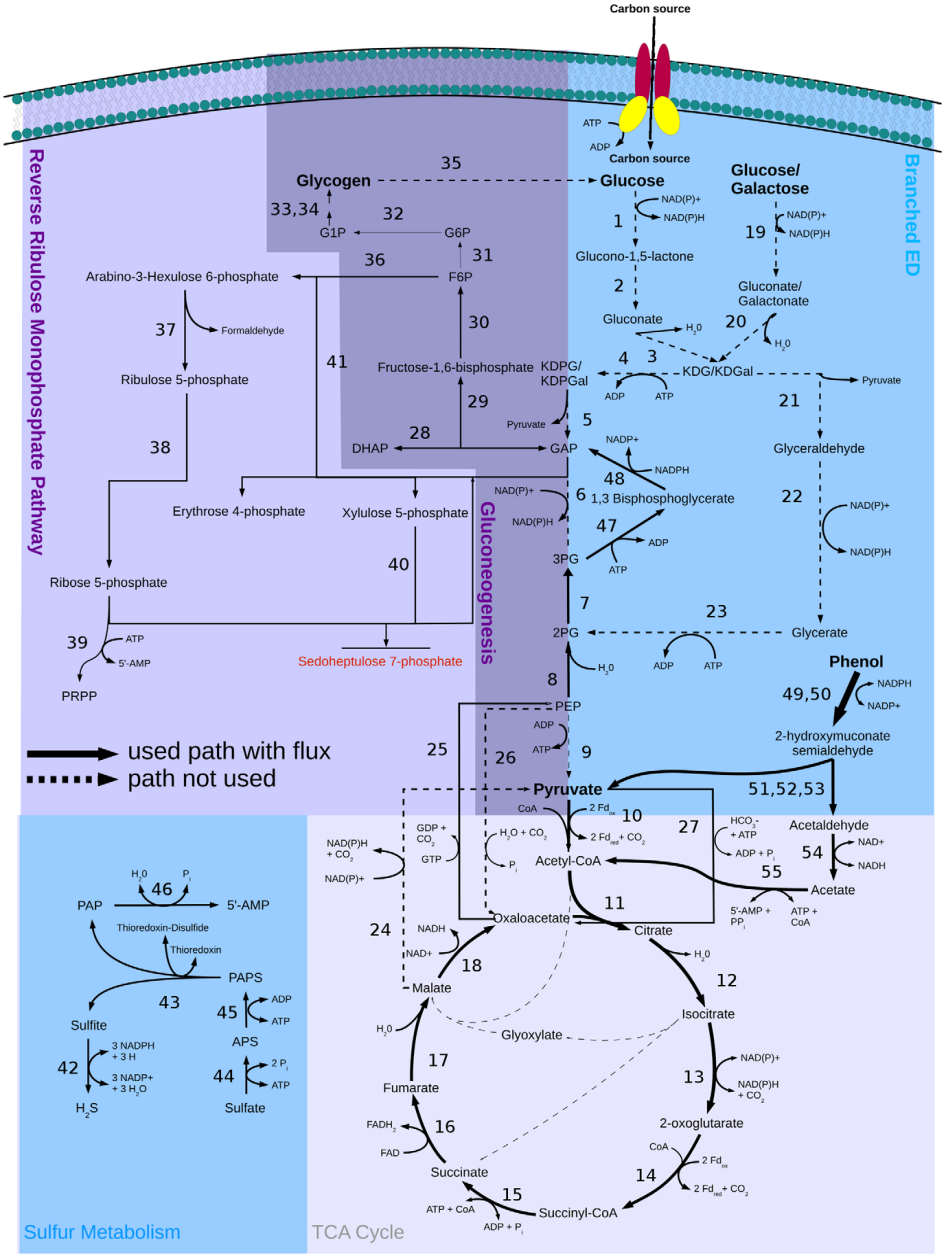
Central carbon metabolism in *S. solfataricus* with flux distribution for growth on phenol. Solid arrows show the used pathways and their directions. Dashed arrows show available but not used pathways. Arrow size represents predicted flux though the pathways. Enzymes in this pathway: (1) glucose 1-dehydrogenase, (2) gluconolactonase, (3) gluconate dehydratase (4) 2-dehydro-3-deoxygluconokinase, (5) 2-dehydro-3-deoxy-phosphogluconate aldolase, (6) glyceraldehyde-3-phosphate dehydrogenase, (7) phosphoglycerate mutase, (8) phosphopyruvate hydratase, (9) pyruvate kinase, (10) pyruvate synthase, (11) citrate (Si)-synthase, (12) aconitate hydratase, (13) isocitrate dehydrogenase, (14) 2-oxoglutarate synthase, (15) succinate-CoA ligase, (16) succinate dehydrogenase, (17) fumarate hydratase, (18) malate dehydrogenase, (19) glucose 1-dehydrogenase, (20) galactonate dehydratase, (21) 2-keto-3-deoxygluconate aldolase, (22) aldehyde dehydrogenase, (23) glycerate kinase, (24) malate dehydrogenase, (25) phosphoenolpyruvate carboxykinase (GTP), (26) phosphoenolpyruvate carboxylase, ((27) pyruvate carboxylase, (28) triose-phosphate isomerase, (29) fructose-bisphosphate aldolase, (30) fructose-bisphosphatase, (31) glucose-6-phosphate isomerase, (32) beta-phosphoglucomutase, (33) glucose-1-phosphate adenylyltransferase, (34) starch synthase, (35) glucan 1,4-alpha-glucosidase, (36) 6-phospho-3-hexuloisomerase, (37) 3-hexulose-6-phosphate synthase, (38) ribose-5-phosphate isomerase, (39) ribose-phosphate diphosphokinase, (40) transketolase, (41) transketolase, (42) sulfite reductase, (43) phosphoadenylyl-sulfate reductase, (44) sulfate adenylyltransferase, (45) adenylyl-sulfate kinase, (46) 3′(2′),5′-bisphosphate nucleotidase, (47) phosphoglycerate kinase, (48) glyceraldehyde-3-phosphate dehydrogenase, (49) phenol 2-monooxygenase, (50) catechol 2,3-dioxygenase, (51) 2-hydroxymuconate-semialdehyde hydrolase, (52) 2-oxopent-4-enoate hydratase, (53) 4-hydroxy-2-ketovalerate aldolase, (54) aldehyde dehydrogenase, (55) acetate-CoA ligase.

Aromatic compounds such as phenol are frequently found in polluted soil. *S. solfataricus* is known to grow on phenol as sole carbon source [16], [37]. To make phenol bioavailable (in *S. solfataricus*), phenol is converted to catechol by phenol 2-monooxygenase (Figure 6: (49)). Catechol is metabolized to pyruvate and acetyl-CoA (Figure 6: (53,55)). Catechol 2,3-dioxygenase (Figure 6: (50)) is the key enzyme in this pathway, opening the aromatic ring. Acetyl-CoA and pyruvate are then used in the TCA cycle for reduction of different coenzymes and for ATP production. Phosphoenolpyruvate, produced by phosphoenolpyruvate carboxykinase (GTP) (Figure 6: (25)), is metabolized by the gluconeogenesis pathway making intermediates available for other pathways such as the reverse ribulose-monophosphate pathway.

In this study, we analyzed the capability of iTU515 to predict aerobic growth on phenol. For comparability, the phenol uptake rate was set to 0.55 mmol g_DW_^−1^ h^−1^ (the same carbon uptake as during growth on glucose), and the NGAM value was kept at the same value as during glucose growth.

**Figure 7:**
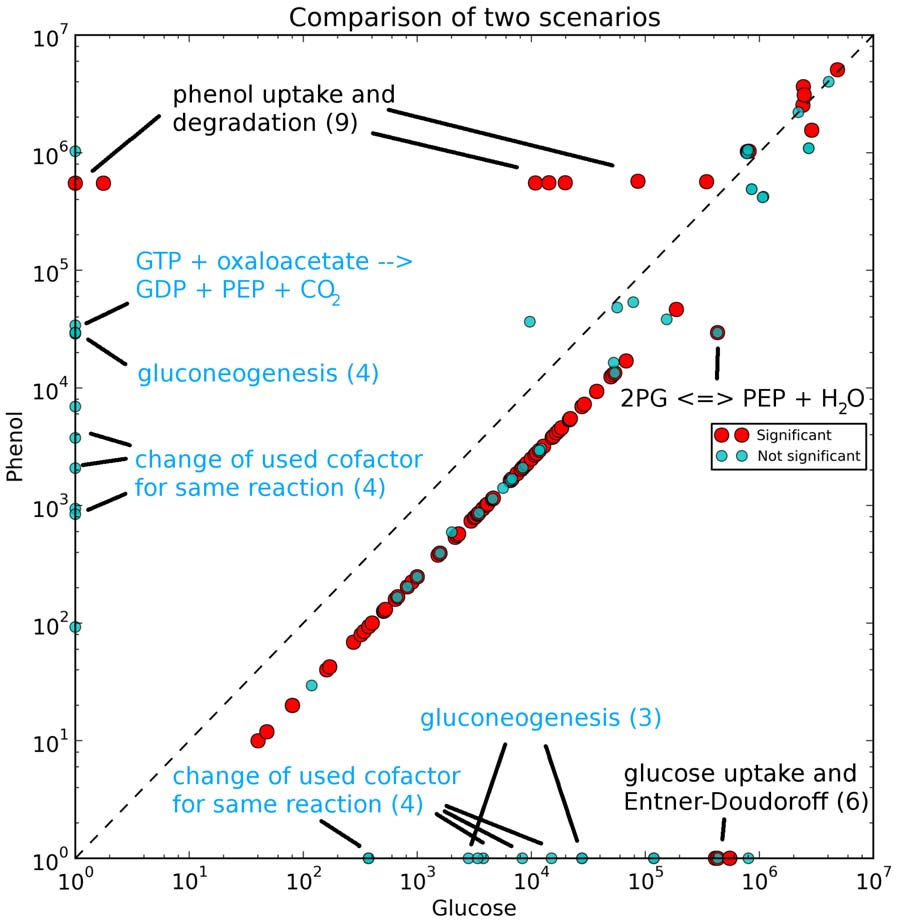
Scatter plot of the flux distributions during growth on glucose and the phenol-utilizing scenario. Red dots show significant differences and blue dots show not significant differences between both scenarios. Blue labels describe not significant differences. The numbers indicate the number of involved reactions.

Figure 7 shows that most of the fluxes are very similar between the two carbon sources. The position of the main diagonal line representing unmodified fluxes is below the main diagonal, reflecting the lower biomass production. Only the active phenol uptake and degradation, the inactive Entner-Doudoroff pathway, and the conversion of 2-phospho-D-glycerate to phosphoenolpyruvate (reversed reaction direction), shared by the Entner-Doudoroff pathway and gluconeogenesis, are significant differences between growth on phenol and growth on glucose. In contrast to growth on glucose, the reaction catalyzed by phosphoenolpyruvate carboxykinase (Figure 6: (25)) is used to ensure the supply of necessary intermediates for gluconeogenesis (oxaloacetate+GTP→phosphoenolpyruvate+GDP+CO_2_) (blue circles; Figure 7). The increased demand of GTP is met by the increased nucleoside-diphosphate kinase reaction. The active gluconeogenesis pathway ensures the supply of necessary intermediates, especially those of the reverse ribulose-monophosphate pathway.

### Growth on different carbon sources

As described above, *S. solfataricus* is able to grow on a variety of organic compounds as carbon and energy sources [10], [49], [50]. For most of them, information about growth rates or identified transporters is not available. The capability of the model to quantitatively predict aerobic growth on various carbon sources was analyzed. For that purpose, FBA was performed with the supply of each carbon source constrained to one millimole of carbon atoms per gram dry weight per hour (e.g. 0.167 mmol g_DW_^−1^ h^−1^ for glucose, 0.25 mmol g_DW_^−1^ h^−1^ for acetoin, 0.5 mmol g_DW_^−1^ h^−1^ for ethanol) [51]. The NGAM flux was set to 0.67 mmol ATP g_DW_^−1^ h^−1^, which corresponds to a carbon usage ratio of 25% for biomass and 75% for respiration for glucose as sole carbon source. This offers a direct comparison of the theoretical efficiency of each carbon source metabolized in iTU515 in contrast to glucose.

The model is able to produce biomass utilizing 35 different carbon sources. For 13 of them the model predicts an increase in biomass production of more than 10% compared to glucose (Table 2).

**Table 2:** Comparison of the achieved biomass flux on 35 different carbon sources in *S. solfataricus*.

Increased flux			Similar flux			Decreased flux		
Carbon Source	Biomass flux	Changed flux (% of glucose)	Carbon Source	Biomass flux	Changed flux (% of glucose)	Carbon Source	Biomass flux	Changed flux (% of glucose)
Glycerol	0.0117	175	Xylose	0.0073	110	Citrate	0.0058	87
Propanol	0.0083	125	Xylan	0.0073	110	Lactate	0.0058	87
Raffinose	0.0082	122	D-Arabinose	0.0073	110	Alanine	0.0058	87
N-acetyl-glutamate	0.0080	120	Arabinan	0.0073	110	L-Arabinose	0.0053	80
Melibiose	0.0079	118	Mannose	0.0070	105	Malate	0.0052	78
Lactose	0.0079	118	Galactose	0.0070	105	Acetoin	0.0052	78
Glutamate	0.0078	118	Glycogen	0.0067	101	Pyruvate	0.0042	62
Trehalose	0.0075	112	Glucose	0.0067	100	Tartrate	0.0040	59
Maltose	0.0075	112	Dextrin	0.0067	100	Ethanol	0.0033	50
Maltodextrin	0.0075	112	Fructose	0.0067	100	Phenol	0.0017	25
Sucrose	0.0075	112	Succinate	0.0065	97	Cytosine	0.0015	22
Cellulose	0.0075	112						
Cellobiose	0.0075	112						

The biomass flux of iTU515 using dextrin and fructose as carbon source showed identical behavior for the fluxes and predicted biomass compared to glucose as carbon source. In addition, 9 other carbon sources showed a biomass yield that differs by less than 10% from that of glucose.

The predicted biomass flux for the remaining 11 carbon sources was decreased on average by 35%. One of the lowest yields (25% of that in the glucose scenario) was observed for phenol. To get a direct comparison of the efficiencies of each carbon source, the energy yield resulting from the conversion of these carbon sources to pyruvate was calculated. Whereas the conversion of phenol to pyruvate needs three NAD(P)H molecules, corresponding to 1.5 ATP, the pathway from glucose to pyruvate, on the other hand, provides a gain of one ATP.

For a better overview, all carbon sources were grouped by their entry point into the central metabolism. The different points of entry are glucose/galactose at the beginning of the Entner-Doudoroff pathway (cellobiose, cellulose, dextrin, glycogen, lactose, maltodextrin, maltose, mannose, melibiose, raffinose, sucrose, and trehalose), glyceraldehyde in the non-phosphorylating branch of the Entner-Doudoroff pathway (glycerol), acetyl-CoA at the link between the Entner-Doudoroff pathway and TCA cycle (acetoin, ethanol, lactate, phenol, pyruvate, and N-acetyl-L-glutamate), and the following TCA cycle intermediates: 2-oxoglutarate (D-arabinose, arabinan, cytosine, L-alanine, L-glutamate, xylose, and xylan), succinyl-CoA (propanol), succinate (succinate), malate (L-arabinose and malate), and oxaloacetate (tartrate).

In summary, most carbon sources that enter the central metabolism through the TCA cycle but not via 2-oxoglutarate (citrate, malate, L-arabinose, acetoin, ethanol, and tartrate) had a significantly lower biomass flux compared to glucose as carbon source. A comparable biomass flux to glucose was achieved by monosaccharides as carbon source, which enter the central metabolism through the Entner-Doudoroff pathway (dextrin, galactose, glycogen, mannose, and fructose) in a similar way as glucose.

A slightly higher biomass yield (110%) compared to growth on glucose could be observed for arabinan, D-arabinose, xylan, and xylose. Two possibilities are described for *S. solfataricus* to degrade these carbon sources [52], with the entry point to the TCA cycle being either 2-oxoglutarate or malate. FBA predicted a higher biomass yield by 30% for the degradation via 2-oxoglutarate. Although these carbon sources do not use the branched Entner-Doudoroff pathway, the biomass flux is slightly increased to that during growth on glucose. Before these carbon sources can enter the TCA cycle, they are metabolized to 2-oxoglutarate reducing 2 NADP^+^. In contrast, glucose metabolism reduces 2 oxidized ferredoxin and 2 NAD(P)^+^ but also consumes 1 ATP for TCA cycle refilling. In terms of energy, 1 ATP is equivalent to 2 NAD(P)H because of the P/O ratio of 0.5. Furthermore, the activity of ferredoxin-NADP^+^ reductase converts 2 reduced ferredoxin to 1 NAD(P)H. From this it follows that the yield from metabolizing arabinan, D-arabinose, xylan, and xylose to 2-oxoglutarate is 2 NAD(P)H in contrast to only 1 NAD(P)H from metabolizing glucose. The difference in energy yield is reduced by comparing the energy needed for the import of one carbon atom of glucose, arabinan, D-arabinose, xylan, or xylose. The import of arabinan, D-arabinose, xylan, or xylose consumes 0.2 ATP per carbon atom, which is slightly higher than the import cost of glucose (0.1667 ATP per carbon atom). A similar biomass flux increase, compared to glucose as carbon source, had already been described for D-arabinose and xylose [53]. In agreement with experiments, FBA predicts an increased activity for malic enzyme, which decarboxylates malate to pyruvate. The induced activity of malic enzyme can be explained by the need of phosphoenolpyruvate for gluconeogenesis. Gluconeogenesis is necessary for the supply of other pathways such as the reverse ribulose-monophosphate pathway.

The last group represents oligosaccharides, which are metabolized via the Entner-Doudoroff pathway like glucose. This group consists of melibiose, sucrose, lactose, cellobiose, trehalose, maltodextrin, raffinose, and maltose. They show an increased biomass flux by 12–22% compared to growth on glucose, although these carbon sources use for the most part the same catabolic reactions like glucose. The explanation is that *S. solfataricus* takes up mono-, di- and oligosaccharides exclusively via ABC transporters [50] which consume one ATP per molecule regardless of the molecule's size. It follows that importing two monosaccharide molecules costs twice as much ATP as importing one disaccharide.

The highest increase in biomass was predicted for glycerol as carbon source. During growth on glycerol the biomass flux increased by 75% in comparison to glucose. Metabolizing glycerol to pyruvate generates two ATP per six carbon atoms in contrast to one ATP gained using glucose as carbon source. In addition, the uptake of glycerol does not consume any ATP, unlike the uptake of glucose via an ABC transporter. This makes glycerol the energetically most efficient carbon source per carbon atom in our study.

In conclusion, compared to glucose most of the carbon sources metabolized via the TCA cycle showed a reduced growth, except for those entering through 2-oxoglutarate. Almost all di- and oligosaccharides showed a slightly higher biomass flux, because of their lower ratio of ATP consumption per imported carbon atom. This information could be used for optimizing the medium composition with respect to biomass gain, but additional comparisons of uptake rates would be necessary. For example, as no experimental value for the ethanol consumption was known and we normalized the simulations to equal carbon atom influx, a decreased biomass flux on ethanol by 50% resulted, which could be easily corrected by assuming a higher ethanol consumption rate [54]. For a more realistic prediction of the growth behavior of iTU515 grown on different carbon sources, it is necessary to determine the natural uptake rates of as many carbon sources as possible.

### Gene essentiality analysis

The essential genes and reactions needed for growth of *S. solfataricus* on glucose were determined using minimization of metabolic adjustment (MOMA) (see Material and Methods). The gene-associated reactions were deleted one gene at a time by constraining the flux through the corresponding reactions to zero. MOMA minimizes the Euclidean distance to the wild type flux vector (FBA). The results of such calculations as presented in the following paragraphs have to be interpreted with caution as the calculations are based on the – necessarily wrong – assumption that all enzymes are expressed. Hence the number of essential genes is underestimated. Transcriptome or proteome data would improve the results.

**Figure 8:**
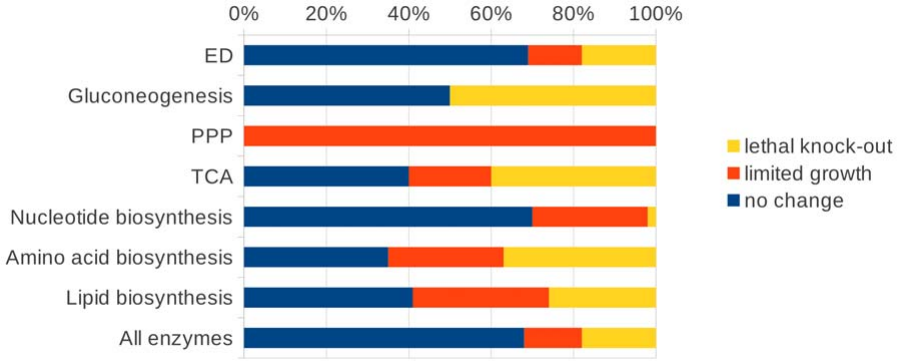
Diagrams of the influence of single-gene deletions in iTU515 on the central carbon metabolism and the whole model (all enzymes) in iTU515.

For 68% of the single-gene deletions, 90–100% of the original biomass flux could be achieved. About 18% of the gene-associated reactions in iTU515 with glucose as carbon source were predicted to be essential. They showed less than 2% of the original biomass flux. About 14% of the tested gene deletions showed a decline in growth to 89–2% of the initial growth. Especially gene deletions in the central metabolism had a highly negative impact on growth (Figure 8).

Thus, for example, each gene deletion in the reverse ribulose-monophosphate pathway was lethal [55], as well as half of the knockouts belonging to gluconeogenesis. This can be explained by the fact that the reverse ribulose-monophosphate pathway represents the only possibility to produce the essential precursors for nucleotide biosynthesis.

Most of the genes related to gluconeogenesis are essential because it leads to fructose 6-phosphate, which is an important precursor leading to the reverse ribulose-monophosphate pathway and the biosynthesis of glycogen and trehalose, which are energy storage molecules.

However, only three of the deletions (genes) in the Entner-Doudoroff pathway were lethal, and two led to a strong decline in biomass formation. In particular, the deletions of gluconate dehydratase (Figure 2: (3)), 2-dehydro-3-deoxy-phosphogluconate (KD(P)G) aldolase (Figure 2: (5,21)), and phosphopyruvate hydratase (Figure 2: (8)) are predicted to be lethal. All of these are particularly important for the connection between glycolysis and the TCA cycle and cannot be bypassed by other reactions.

In the TCA cycle a total of six deletions resulted in no or reduced biomass flux. Knockouts are lethal (four reactions) when reactions cannot be circumvented via other pathways (e.g. citrate synthase (Figure 2: (11)), aconitase (NAD(P)^+^) (two reactions; Figure 2: (12)), and fumarase (Figure 2: (17))).

In the amino acid and lipid metabolism, 37% and 26% of the knockouts were lethal, respectively.

## Conclusion

To our knowledge, iTU515 is the first genome-scale reconstruction of *Sulfolobus solfataricus* P2, a hyperthermoacidophilic Archaeon. We have developed a manually and automatically curated genome-scale constraint-based model of the metabolism of *S. solfataricus* to achieve high agreement between iTU515 predictions and experimental findings. iTU515 represents a comprehensive knowledge base summarizing the information currently available for *S. solfataricus*. Moreover, the model iTU515 is a highly detailed reconstruction of the metabolic network of *S. solfataricus*, which includes important biotechnological capabilities, such as the degradation of phenol as sole carbon source. We also analyzed the biomass yield of 35 different carbon sources compared to glucose. We discovered glycerol to be the single carbon sources with the highest biomass flux per carbon atom in our study. Moreover, we determined (i) the flux distribution through the network in different scenarios and compared distributions between scenarios, (ii) the active reactions and pathways, (iii) the variability of the network under the constraint of (sub)optimal biomass yield and, (iv) the robustness of the network against gene deletions.

## Materials and Methods

### Model reconstruction

The main reconstruction of the initial *S. solfataricus* model was done manually (Figure S1) starting with an annotation of the genome compiled by the software tool EnzymeDetector [56], which predicted 2,977 EC numbers with different annotation quality scores, represented in a web-based and in a database format. For the genome-wide reconstruction, we used the annotation from EnzymeDetector; different databases including KEGG [18], MetaCyc [19], a *Sulfolobus*-specific annotation database [14], and BRENDA [20]; as well as the primary literature. At the end of the reconstruction process, the model was extended automatically by new reactions using a self-written Python program (see below). During this procedure, any remaining reactions corresponding to annotated but not integrated enzyme classes were added to the final model.

Manual model building started with the core of the network, composed of the central carbon metabolism (CCM) and essential transporters, namely those for glucose, water, CO_2_, O_2_, protons, and phosphate. We reconstructed the CCM using literature and biochemical databases. Afterwards, the core model was extended by adding new pathways consecutively. Whenever a reaction was added to the network, it was checked whether all reactants of each new added reaction were already present in model. For each newly-introduced reactant, a transport reaction for the import or export was added. This procedure was repeated until each compound of the biomass (vide infra) and all cofactors could be reached and all artificial transporters could be removed. Organism specificity of the reactions was achieved by manually including unique metabolites and pathways known to be present in *S. solfataricus*, such as the unusual branched Entner-Doudoroff pathway [48], [57], the Embden-Meyerhof-Parnas (EMP) [44] pathway, which is employed during gluconeogenesis, the reverse ribulose-monophosphate pathway, which replaces the missing oxidative pentose phosphate pathway, the unique sulfopterin biosynthesis [58], and the biosynthesis of the coenzymes caldariellaquinone and sulfolobusquinone [59], [60]. Moreover, the measured stoichiometric values for proton translocation in the electron transport chain were included [25], [61].

Transport reactions were added from the database TransportDB [62] or alternatively from physiological data. Gaps occurring in added pathways were closed by postulating EC numbers if there was evidence for the presence of the respective pathway in *S. solfataricus*. Evidence for the postulated enzyme classes was gathered from literature using a hierarchical approach starting at the species level. If no species-specific evidence was found, the search was extended to the genus and finally to the domain level.

Finally, the model was extended automatically. If an enzyme function was predicted by the annotation, the EC number and the respective reaction were automatically included. Reaction stoichiometry and reversibility were checked before any reaction was added to the network. Information on reaction reversibility was primarily gained from literature and further from BRENDA. Additionally, thermodynamic considerations were used for determining reaction reversibility and direction in case of irreversibility. For example, reactions consuming high-energy metabolites such as ATP and carbon-dioxide-producing reactions are generally irreversible, with the exception of ATP-producing and anaplerotic reactions.

### Biomass composition

The biomass reaction was used as the objective function. Maximization of the flux through the biomass reaction simulates a cell which has been optimized by evolution for optimal usage of resources and efficient growth. A detailed biomass composition for cells growing in the log phase was not available. Therefore, the relative biomass composition of *Methanosarcina barkeri* [7], a different archaeon, was used [29]. The molar percentage and molecular weight of each monomer was used to calculate the weight per mole protein, RNA, and DNA, based on the data from the *S. solfataricus* genome (http://cmr.jcvi.org) [28]. Subsequently, all the individual values of all amino acids (protein) and all nucleotides (RNA, DNA) were added to get a total molecular weight of the protein, RNA and DNA content, respectively. The biomass reaction sums the molar proportions necessary to produce the cellular growth per gram dry weight per hour (mmol g_DW_^−1^ h^−1^). The molar contribution of the other components such as carbohydrates, lipids, and the contents of the soluble pool (polyamines, vitamins, and cofactors) of the *S. solfataricus* biomass reaction were taken from the biomass reaction of *M. barkeri* [7] and primary literature [14], [63].

### Genome annotation

A weighted combined annotation of the *Sulfolobus solfataricus* P2 [14], [64] genome was constructed using the in-house software EnzymeDetector. This software automatically integrates the predicted function for each enzyme class from the main annotation databases and adds its own annotation [65] based on sequence similarity and sequence patterns (BrEPS [66]). Additionally, EnzymeDetector searches for organism-specific enzyme activities in BRENDA. Also the organism-specific database of the “*Sulfolobus solfataricus P2 complete genome sequencing project*” [14] was included in the search to improve the quality of the annotation. This way, the program provides a fast and up-to-date compilation of the available information for the organism under consideration using user-definable weighting schemes and acceptance thresholds. These results were then divided into the following levels of annotation quality: low, middle, high, and experimentally verified (biological). Entries with low relevance are those that are only predicted by one annotation source and/or have a comparably high E-value. The predicted EC numbers in the high-relevance group are present in almost all databases and have a good E-value. All enzyme classes were assigned a very high relevance score of “biological” when they were experimentally determined by our lab to be expressed in *S. solfataricus* or found in the literature (included in BRENDA and/or AMENDA).

A rating system for the quality of enzymes included in the model was employed to rate each reaction in the network with regard to its presence in *S. solfataricus* as predicted by EnzymeDetector. The rating system is represented by a self-defined confidence score (1–4), which was used in this work. Those reactions and EC numbers that have been biochemically proven to be present in *S. solfataricus* received a confidence score of 4, including the EC numbers found in BRENDA for *S. solfataricus*. A score of 3 marked an EC number when the prediction is based on high BLAST (EnzymeDetector) evidence (E-value<10^−80^) and the function was also predicted by most of the main annotation databases. Reactions were assigned a confidence score of 2 when the BLAST search found a homologous enzyme with an E-value>10^−40^ for this function and when this EC number was also predicted by some of the main annotation databases. During the model reconstruction phase some reactions had to be added to the network to fill gaps. For these enzymes no genetic or experimental evidence was found, or they were found only with a very bad E-value by the BLAST search. They were marked with a confidence score of 1. The final reconstructed network has an average confidence score of 2.75. 35% of all *S. solfataricus* reactions are experimentally verified using the data from BRENDA and AMENDA (evidence score of 4). Over 21% of the reactions in the network have an evidence score of 3. Enzymes of these reactions have very good genome-based evidence. 28% of the reactions are based on a good genome annotation (2). Only the remaining 16% are based on a low genome annotation quality or had to be included without genome evidence (1). These reactions belong to pathways for the degradation of some carbon sources (e.g. tartrate, phenol) or biosynthesis of coenzymes like vitamin B_12_. The explanation is that there are published results that *S. solfataricus* is able to grow on several carbon sources or to produce the coenzymes by itself, but there is no evidence in the genome for the necessary enzymes.

### *In-silico* flux prediction using flux balance analysis

The open-source software toolbox *metano* (available from http://metano.tu-bs.de/; Riemer et al., manuscript in preparation) was used for the computations in this work. *metano* natively uses a human-readable ASCII format for model input, but is also able to import SBML files [67]. Underlying flux balance analysis (FBA) [4], [5], [68] is the assumption that organisms are optimized through evolution. Here maximum growth was used as the objective function. A stoichiometric matrix **S** (*m*×*n*) is used, where each row corresponds to a metabolite and each column to a reaction. A positive entry *S_i_*_,*j*_ indicates that compound *i* appears on the right-hand side in reaction *j*, while a negative value means that it appears on the left-hand side. The change in metabolite concentrations is described by this equation:
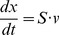
(1)i.e. the matrix S is a linear transformation of a flux vector **v** = (*v*_1_, *v*_2_, …,*v_n_*)^T^ to a vector of time derivatives of the concentration vector **x** = (*x*_1_, *x*_2_, …, *x_m_*)^T^. In steady state, all metabolite concentrations in the system under consideration are constant:
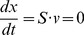
(2)Any **v** that satisfies equation (2) lies within the solution space. The solution space is further constrained by inequalities of the form *v_i_*_,min_≤*v_i_*≤*v_i_*_,max_ for each reaction *i*, where *v_i_*_,min_ and *v_i_*_,max_ are lower and upper bounds of the reaction flux. Reversibility and irreversibility of reactions are defined by the constraints −∞≤*v_i_*≤∞ and 0≤*v_i_*≤∞, respectively. Fluxes can be further constrained by experimental results. The uptake of a metabolite was defined as 0≤*v_i_*≤∞, and secretion of a metabolite was defined as −∞≤*v_i_*≤0 for every exchange reaction *i*. These reactions define an *in-silico* bioreactor where the exchange metabolite is continuously added to a virtual medium, and secreted products are continuously removed. A metabolic network typically has more reactions than metabolites (*n*_>_ *m*). This implies that the system of linear equations (2) is underdetermined, and the solution is not unique.

Finally, the use of constraints corresponding to observed environmental conditions (e.g. minimal medium, secretion) or different genetic conditions (e.g. knockout mutants) leads to an organism-specific model.

### Flux variability analysis

Metabolic networks of living organisms are usually underdetermined, as described above. Because of the linear nature of the FBA problem, it is easy to determine a minimum and a maximum value for each constrained flux variable *v_i_* such that the biomass flux is still optimal. Flux variability analysis (FVA) [69] computes these values by separately minimizing and maximizing each flux variable *v_i_* under the added constraint that the biomass flux be above some threshold. Artificial constraints |*v_i_*|_<_100,000 are added for unconstrained fluxes. The difference *v_i_*_,max,FVA_ – *v_i_*_,min,FVA_ is a measure for the flexibility of *v_i_* with respect to the goal of maximum biomass production.

Because of the artificial assumption of a fully optimized biomass production, the incompleteness of the network, and the fact that not all enzymes coded on the genome are expressed in a given condition, there is no perfect fit between real organisms and computed results. Suboptimal FVA can be applied where instead of fixing the biomass reaction flux to its optimum, the biomass reaction flux is allowed to vary within a certain range. The lower limit accepted for the biomass reaction flux used in this work was set to 95% of the optimum. The threshold of 95% was chosen to show that a slight change towards achieving an optimal solution can have a big effect on the variability of the fluxes in the network.

An FVA calculation provides numerical values (variability intervals) for each of the predicted fluxes signifying how well a flux value is defined under the given constraints, i.e. the size of the solution space in the direction of this flux. Comparison of the variability intervals of the same reaction computed by FVA in different growth scenarios, e.g. growth on glucose vs. growth on phenol, allows the identification of significant differences. Variability intervals that do not overlap identify significant differences because the corresponding reactions will never assume the same flux.

### Gene essentiality analysis

An *in-silico* knockout study was performed in order to determine the effects of all single-gene deletions on the model. A simple approach is to sequentially set the flux through each gene-associated reaction to zero and perform an FBA calculation with this additional constraint. This method only determines if a gene deletion is lethal or not. If no positive biomass flux is obtained, the deletion of the reaction is by definition lethal.

To find out how the network behaves to perturbations by single-reaction deletion, the MOMA method (Minimization of Metabolic Adjustment) [70] was applied. MOMA is based on the assumption that the fluxes of a perturbed network undergo a minimal redistribution with respect to the fluxes of the wild-type network. This is a realistic approach because mutant organisms have not undergone an evolutionary optimization in contrast to the wild-type. MOMA determines the flux vector with a minimal Euclidean distance to the wild-type flux vector (FBA). This analysis yields a more realistic *in-silico* prediction of the fluxes in the metabolic network of a mutant.

## Supporting Information

Figure S1 **The iterative and automatic model building procedure used to generate iTU515.** The genome annotation was used as a scaffold for the genome-scale model. The reactions added to the model were taken from both biochemical databases and published data. FBA simulations under steady-state conditions were used to determine the reaction flux distribution in the network and to improve the model.
(TIFF)
