# Supplementary figures and images for: Genome-Scale Reconstruction and Analysis of the Metabolic Network in the Hyperthermophilic Archaeon Sulfolobus Solfataricus
Source: PLoS One. 2012 Aug 31;7(8):e43401. doi: 10.1371/journal.pone.0043401 (PMC3432047; doi:10.1371/journal.pone.0043401)

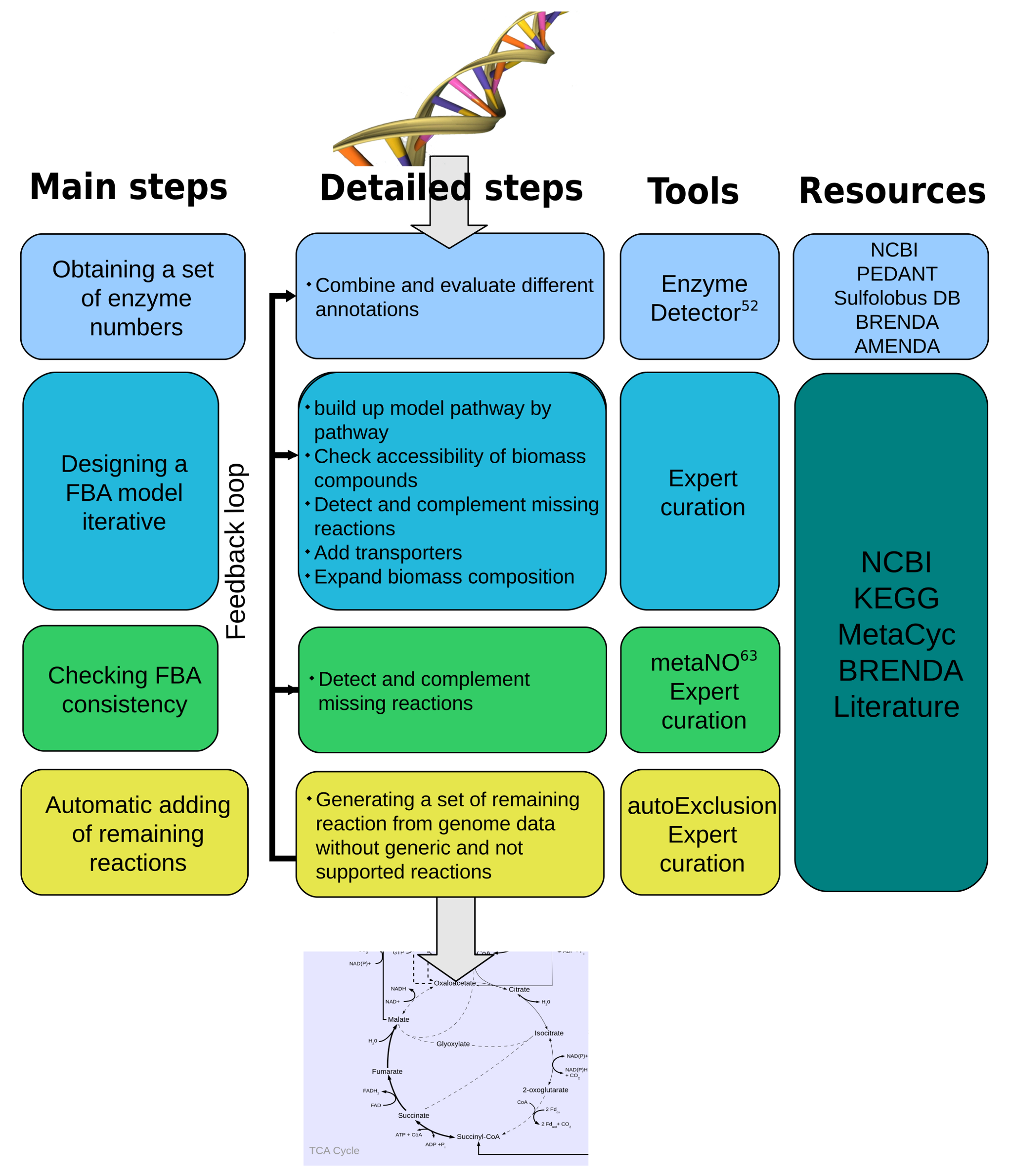

Supplement: Figure S1 — The iterative and automatic model building procedure used to generate iTU515. The genome annotation was used as a scaffold for the genome-scale model. The reactions added to the model were taken from both biochemical databases and published data. FBA simulations under steady-state conditions were used to determine the reaction flux distribution in the network and to improve the model. (TIFF) [file pone.0043401.s001.tiff]
